# Local chatter or international buzz? Language differences on posts about Zika research on Twitter and Facebook

**DOI:** 10.1371/journal.pone.0190482

**Published:** 2018-01-05

**Authors:** Germana Barata, Kenneth Shores, Juan Pablo Alperin

**Affiliations:** 1 Laboratory of Advanced Studies in Journalism, State University of Campinas (Unicamp), Campinas, SP, Brazil; 2 Scholarly Communications Lab, School of Publishing, Simon Fraser University, Vancouver, BC, Canada; 3 Graduate School of Education, University of Pennsylvania, Philadelphia, PA, United States of America; Tampere University of Technology, FINLAND

## Abstract

**Background:**

When the Zika virus outbreak became a global health emergency in early 2016, the scientific community responded with an increased output of Zika-related research. This upsurge in research naturally made its way into academic journals along with editorials, news, and reports. However, it is not yet known how or whether these scholarly communications were distributed to the populations most affected by Zika.

**Methodology/Principal findings:**

To understand how scientific outputs about Zika reached global and local audiences, we collected Tweets and Facebook posts that linked to Zika-related research in the first six months of 2016. Using a language detection algorithm, we found that up to 90% of Twitter and 76% of Facebook posts are in English. However, when none of the authors of the scholarly article are from English-speaking countries, posts on both social media are less likely to be in English. The effect is most pronounced on Facebook, where the likelihood of posting in English is between 11 and 16% lower when none of the authors are from English-speaking countries, as compared to when some or all are. Similarly, posts about papers written with a Brazilian author are 13% more likely to be in Portuguese on Facebook than when made on Twitter.

**Conclusions/Significance:**

Our main conclusion is that scholarly communication on Twitter and Facebook of Zika-related research is dominated by English, despite Brazil being the epicenter of the Zika epidemic. This result suggests that scholarly findings about the Zika virus are unlikely to be distributed directly to relevant populations through these popular online mediums. Nevertheless, there are differences between platforms. Compared to Twitter, scholarly communication on Facebook is more likely to be in the language of an author’s country. The Zika outbreak provides a useful case-study for understanding how scientific outputs are communicated to relevant populations. Our results suggest that Facebook is a more effective channel than Twitter, if communication is desired to be in the native language of the affected country. Further research should explore how local media—such as governmental websites, newspapers and magazines, as well as television and radio—disseminate scholarly publication.

## Introduction

The Zika virus has been known to affect humans since 1952, but in February 2016 it was declared a “Public Health Emergency of International Concern” by the World Health Organization [1]. The outbreak received international attention, in part because one of the most affected countries, Brazil, was about to host the Olympic and Paralympic Games that August. The impending mega-event and the influx of tourists it would attract from around the world turned the Zika outbreak from a national issue into a global health threat.

The emerging nature of the outbreak, the seriousness of the disease, and the global public interest on the topic all demanded accurate information to mitigate the spread of the virus and to effectively treat those affected. Driven by the urgency to respond to Zika, funding agencies made resources available to researchers [2,3] and scientific journals attempted to accelerate science communication by opening up fast track papers (accelerating editorial decisions, peer review and publication) [4–6], providing room for other document types such as editorials, news and social media, as well as making the research freely available through open access publishing [7,8]. The result was nearly a 3,000 percent increase in papers with “Zika” in the title since the beginning of 2016, as seen on the Scopus database [9].

As the number of infected people increased dramatically, researchers, public officials, and science communicators also turned to social media to disseminate information within the scientific community and beyond. With a larger and growing user base, social media platforms have become a powerful way to inform and influence people, and a strategical tool to reach society on public health issues [10,11]. Increasingly, health experts and practitioners are realizing that “one fact sheet or an emergency message about an outbreak can be spread through Twitter faster than any influenza virus” [12]. As such, it is important to recognize the potential of social media to contribute to the improvement of health outcomes and to influence health policy [13].

Considering that academic journals are the scientific community’s formal channel for scientific communication, this study describes how journal’ content on the Zika virus are discussed on social media during a time when reliable information needed to be diffused rapidly to advance research and inform both policy makers and the public at large. In particular, this study investigates the activity around journal articles about Zika on two social media platforms: Facebook and Twitter. Both platforms are important, although for different reasons. Facebook is the most popular social media platform worldwide for sharing, reading, watching and finding news [14] with more than 2 billion users [15]. While Twitter has a smaller reach, with 328 million users [16], it has become a prominent area for scholarly debate [17]. By analyzing the documents about Zika shared on these two social media platforms, we seek to better understand the uptake of scientific community’s efforts to relay reliable information about the emerging outbreak.

Of special interest is whether those scientific outputs are reaching local populations (who are more directly affected by the outbreak), and the factors that influence where and how the research is discussed. Thus, the objective of this study is to determine whether *research articles about Zika shared on Facebook and Twitter reached both national and international audiences*, *as observed through the use of English and local languages*. More specifically it asks the following research questions:

**RQ1:** What languages are used when sharing Zika research on Facebook and Twitter? Does the language used differ between the two platforms in ways that suggest one is used to reach more local or more international audiences?

**RQ2:** Does the author’s country affiliation affect what languages are used in social media posts about Zika research?

In answering these questions, this paper seeks to contribute to a better comprehension of how national and international research takes place on social media by using the Zika outbreak as an example. Considering the period when Zika outbreak achieved its peak, from January to June 2016, our results may also contribute to improve communication strategies of scientific information during crucial periods.

## Previous research

Even though social media users are not representative of the whole population [18], discussions on social media can reflect conversations about health issues and might provide important data for public health surveillance. In fact, social media has already been used to forecast Zika outbreaks, with some models predicting incidence rates up to a week ahead of official public releases [19].

Other studies have also sought to document social media activity related to Zika [20–23]. Stefanidis et al. [22] showed that between December 2015 and March 2016, Tweets about Zika changed from a local to a global concern, with Brazil and Colombia as the first nations to spread tweets about Zika. Their results suggest that there was both a local and a global concern about Zika, and not simply a single global conversation regarding the outbreak. This is affirmed by the work Fu et al. [20], who found that before the outbreak was declared a global health threat, conversations about Zika were dominated by Spanish and Portuguese, but by February 2016, English and Spanish began to dominate in similar proportions. The authors highlight the “need of multilingual Twitter health communication on ZIKV [Zika]” (p. 1702), although their subsequent analysis focuses exclusively on tweets in English. While Sharma and colleagues [23] concluded that Facebook is an important platform for health dissemination, yet revealing that misleading videos about zika where way more popular than the accurate information about the disease, which is particularly crucial to health authorities to take action on changing this scenario. While Miller et al. [21] also targeted misleading information shared on Twitter by automated content classification tool as a way to intervene and provide policy responses to information about Zika.

The limited language analysis done by Stefanidis et al. [22] suggests that language can be used to identify engagement on the local level, while posting in English might indicate an intent to reach global readership. Although social media platforms are typically considered global communication tools that can be used to reach any internet user, individuals may use them with the intent to reach smaller or more local and regional groups. Belling and de Bres [24] analyzed the use of four languages in a multilingual Facebook group, showing how the group started using English as the lingua franca, but over time users moved towards using their local languages. Multilingualism also plays a role in connecting users, as bilingual users act as a key bridge across transnational networks [25,26]. More broadly, Weerkamp et al. [27] have verified that there are cultural differences in how users make use of a platform, as users who speak different languages make different uses of Twitter’s affordances (e.g., use of links, length of tweets, number of retweets, etc.).

While the above studies have been useful for understanding the nature and extent of the general public’s interest in Zika, they have not analyzed the spread of reliable scientific knowledge in academic papers on social media, nor the language choice of those users who are propagating the research.

## Methods

Social media mentions of scientific journal articles related to Zika were based on a local copy of the database from Altmetric LLC, a company tracking online activity around scholarly research outputs with Digital Object Identifiers (DOIs) since 2011. We identified Zika-related documents by searching for “Zika” in the title field and restricted Facebook and Twitter posts to the first six months of 2016 (January 1^st^, to June 30^th^, 2016), the period immediately before and after the outbreak, when information was most scarce). This period also corresponds to the peak of Zika cases [28] as well as the main internet users interest, according to Google Trends [29,30].

Although Altmetric LLC collects information about documents shared on a variety of social media platforms, we only included documents that had been shared on either Facebook or Twitter at least once. The resulting search yielded 844 documents, of which we eliminated 126 that were deemed to be from publications that are not typically considered scholarly journals (i.e., *The Conversation*, *The Winnower*, *Figshare*, *Journal Watch*, and others), 1 that was not a traditional scholarly output (a podcast from *American Journal of Perinatology*), and 5 that were not actually about Zika (e.g. documents that contained the character string “zika” in a different context, such as in relation to the amphipod *Wangiannachiltonia guzikae*). After filtering out these “non-journals” (154), “non-papers” (1) and “non-Zika” (5) documents, the resulting dataset contained 718 documents. We should note that we opted to include 87 documents from the preprint repositories arXiv (4), bioXiv (36), as well as the MMWR: Morbidity & Mortality Weekly Report from Centers of Disease Control and Prevention (CDC) (47). Some CDC reports are “early versions,” which we include as separate documents, since they receive unique identifiers and each appear at a unique internet address. These early reports are similar to preprints, which may contain differences between the original and the final published version. Although not traditionally considered journals, preprints and reports from CDC do typically contain original research results and other strategic information for public health matters [31]. By accessing each article manually, we identified the language of the document, the country of the authors’ stated affiliations, and the country of the journal’s publication. The latter was determined by looking at the mailing address on the journal’s website, or by the country affiliation according to Scimago Journal and Country Rank, when available. For each of the countries, a determination was made if English was commonly used or recognized as an official language.

Documents from our sample were collectively tweeted 43,211 times and shared on public Facebook group or institutional pages 2,307 times. Restricting social media activity to the first six month of 2016, reduces the dataset to 42,705 tweets and 2,275 Facebook posts (almost 99% of all the activity). This highlights that discussions on social media were most intense during the selected period. Since our interest was in studying the language of the social media posts about these articles, we ran a language detection algorithm on the text of each of the tweets and Facebook public page posts. After removing URLs, hashtags (both the *#* and the text associated with it), and @mentions, which are not considered part of the actual post content, using the python module *twitter-preprocessor*, the remaining text was put through python module *langdetect*, a language detection library ported from Google’s language-detection code. To verify the effectiveness of the automated language detection, we manually checked a random selection of 100 tweets and 100 Facebook posts and found the algorithmic detection disagreed with our manual evaluation less than 2% of the time. In a few cases removing the Twitter affordances described above left little or no text leading the algorithm to fail to detect a language for 271 tweets and 1 Facebook post.

We then observed the differences in languages used when posting about research articles on both social media platforms by calculating the number and percentage of posts in each language, across the two platforms, and between the most common author countries (USA, Brazil, UK, and France, in descending order) and their corresponding languages (English, Portuguese, and French), plus Spanish, given its similarity to Portuguese.

To test the significance of these differences, we estimate the probability of whether a social media post will be in English or Portuguese if the author’s country is either the USA, the UK, Brazil or France. Probabilities are taken from a logistic model of the form:

$$Pr(Y_{i}=1|X)=F(\alpha +\beta_{1}Twitter+\varepsilon_{i})$$ (1)

Where *Y*_*i*_ represents whether a social media post in English or Portuguese. *Twitter* is an indicator variable equal to one if the social media outlet was Twitter (the omitted category is Facebook). We estimate the model for different sub-populations, limiting the data to whether at least one author was from the USA, the UK, Brazil or France. In total, we estimate 8 models (2 outcomes and 4 author countries). To ease interpretation, we convert the log-odds into probabilities. Probabilities are equivalent to the difference in the exponential values of β_1_when *Twitter* is set equal to 1 and 0 (i.e., e^β1(Twitter|Twitter = 1)—β1(Twitter|Twitter = 0)^).

We subsequently test if social media posts differ based on whether the author’s country of origin is a primarily English-speaking country. This model has the advantage of including all of the data we collected (i.e., not limiting to those with papers from the four countries of focus). We generate three indicator variables: the variable *No Authors English* (the omitted category) is equal to 1 if none of the authors are from a country where English is the primary language (0 otherwise). The variable *Some Authors English* is equal to 1 if at least one but not all of the authors are from a country where English is the primary language (0 otherwise). The variable *All Authors English* is equal to 1 if all the authors are from a country where English is primary. We then estimate a model of the form:

$$Pr(Y_{i}=1|X)=F(\alpha +\beta_{1}\;Twitter\times (\beta_{2}\;No\;Authors+\beta_{3}\;Some\;Authors+\beta_{4}\;All\;Authors)+\varepsilon_{i}).$$ (2)

The outcome *Y*_*i*_ is equal to 1 if the post was in English and 0 otherwise. The quantities of interest are the marginal probabilities of posting in English on Twitter if some or all of the authors are from an English-speaking country relative to posting in English on Twitter if none of the authors are affiliated with an English-speaking country. These marginal probabilities are calculated as е ^(β1*β3)-(β1*β2)^ and e ^(β1*β4)-(β1*β2)^.

## Results

The majority of the research articles that were eventually shared on social media during the period studied were published in English. Of the 718 in the dataset, 648 were only available in English, and an additional 9 were in English as well as a second language. The next most popular language was Portuguese, with only 4 documents exclusively available in that language, and an additional 6 published in both Portuguese and either Spanish or English. This dominance of English is also reflected in the countries of the journals where these documents are published, which have 330 (46.0%) published in the US and 235 (32.7%) published in the UK. Only 25 (3.5%) of the articles that were shared on social media were published in Brazilian journals.

**Fig 1:**
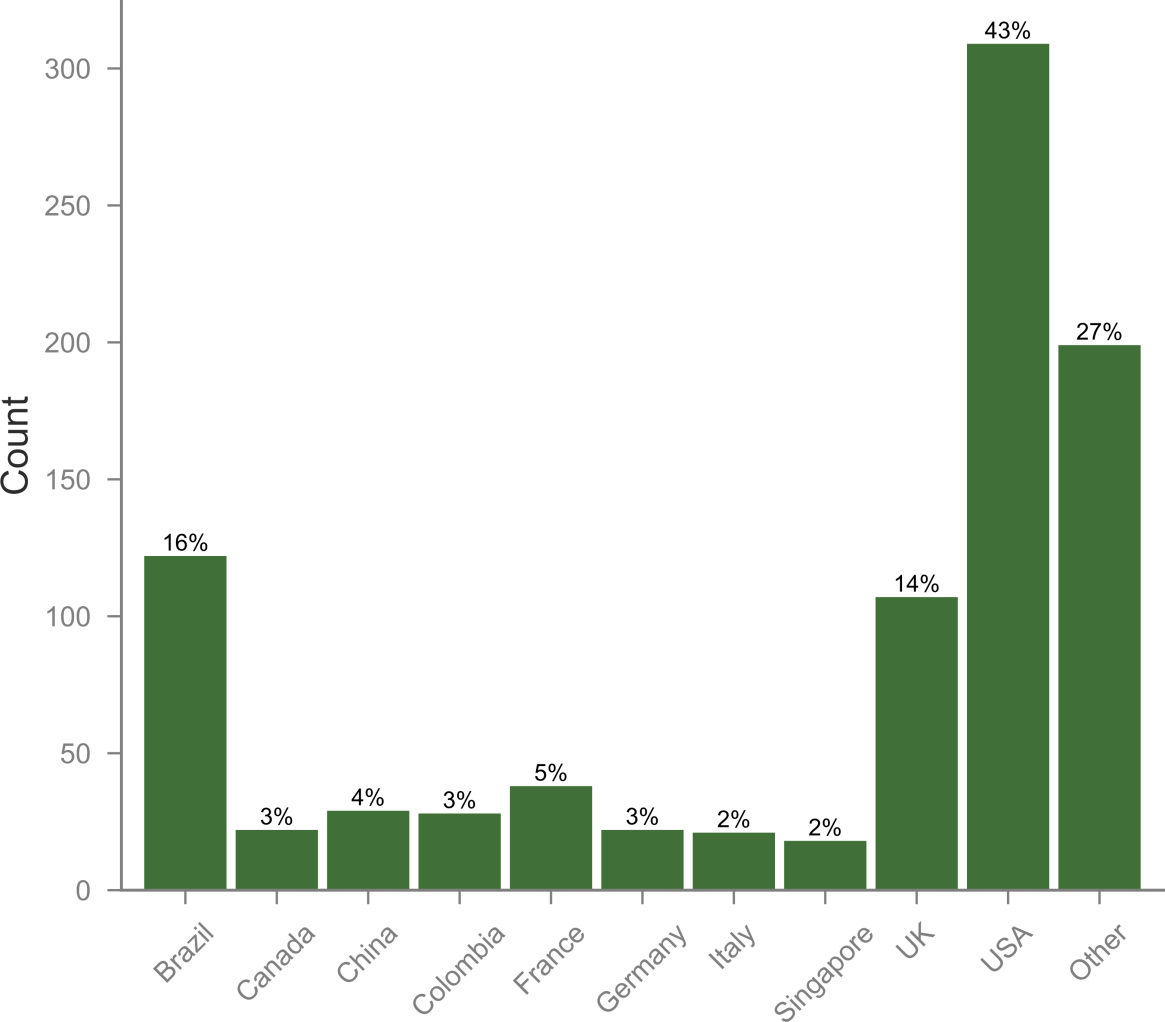
Top countries whose authors had their Zika research shared on social media. Note: Sum of percentages exceeds 100% due to double-counting publications co-authored by more than one country.

**Fig 2:**
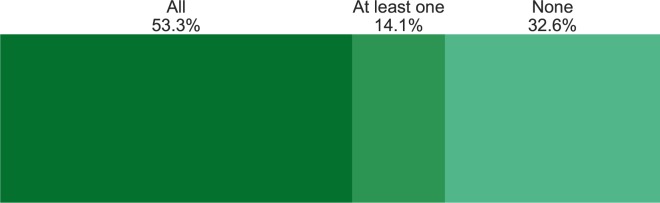
Percent of papers with authors from all, some, or no countries that consider English the main language.

Despite this clear preference for items written in English and published in US and UK-based journals (collectively publishing 78.7% of all the articles), the content of those documents is coming from authors from a much broader range of countries. Although US-based authors remain the most commonly found among these papers (with 43.0% of all papers having at least one US-based author), at least one Brazilian author is found on 17.0% of the articles (Fig 1). It is notable that although 98.1% of the papers were written in English, 35.2% of them were written exclusively by authors from countries who do not use English as an official language (another 14.1% had at least one author from a non-English speaking country) (Fig 2).

**Fig 3:**
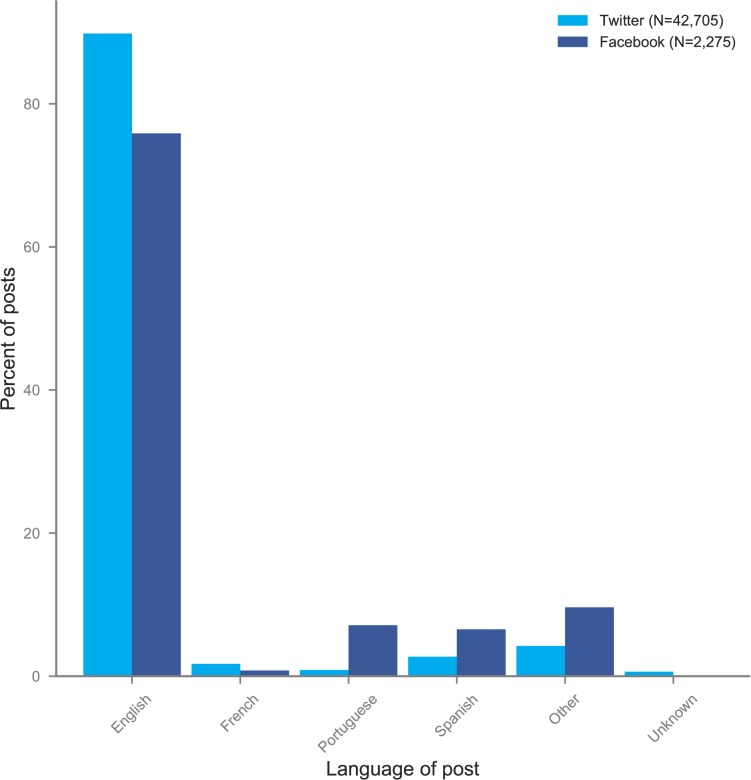
Proportion of social media posts in select languages about Zika-related papers.

Although the preferred language of communication for the scientific community is English, social media offers an opportunity to observe the languages in which the research is being shared and discussed. We observe substantial differences in the languages used between Twitter and Facebook, with 89.8% of tweets but only 75.9% of Facebook posts written in English (Fig 3). On Facebook, we observe much higher use of Portuguese and Spanish (each around 7%), neither of which surpasses 3% of posts on Twitter.

Disaggregating the language used on social media by the country of the author of the scholarly article, we continue to see a greater use of English regardless of the author’s country, with Spanish as the second most used language (Table 1). Despite the importance of Zika in Brazil, and the prevalence of Brazilian authors overall among Zika publications (second only to authors from the USA), we find little use of Portuguese on Twitter (0.8% of all tweets, and 1.5% of tweets about papers with Brazilian authors). Papers with Brazilian authors did have the highest proportion of tweets in languages other (6.2%) than the top four English, Spanish, French and Portuguese.

**Table 1:** Proportion of Twitter posts in most select languages by article author country.

Author Country	Twitter Post Language						N
	English	Spanish	Portuguese	French	Other	Unknown	
USA	91.3%	2.6%	0.4%	1.7%	3.4%	0.6%	24,134
Brazil	88.8%	3.1%	1.4%	1.5%	4.4%	0.8%	7,220
UK	85.9%	3.2%	1.8%	2.3%	6.2%	0.5%	7,797
France	90.4%	2.9%	0.7%	1.8%	3.7%	0.6%	3,011
Other	89.9%	2.8%	0.9%	1.1%	4.7%	0.7%	12,947

On Facebook, we find a different use of languages, with a still dominant but decreased use of English in comparison to Twitter and a greater use of Spanish, Portuguese and other languages across all author countries (Table 2). In fact, on Facebook, we see a more pronounced ‘local’ effect, with increased proportions of posts in the language that matches the language spoken in the author’s country. For example, 13.9% of Facebook messages are in Portuguese if at least one of the author’s is from Brazil; and we see more messages in French (3.9%) if at least one of the authors is from France than any other author country. Although, it should be said that after English, papers from France were talked about in Spanish, Portuguese more often than in French (7.8% and 9.2% of posts in Spanish and Portuguese respectively, versus 3.9% in French). Papers from authors from countries other than the top four were talked about in Spanish and Portuguese in similar proportions (8.1% and 8.6%) and in languages other than the four selected in 9.2% of posts.

**Table 2:** Proportion of Facebook posts in select languages by article author country.

Author Country	Facebook Post Language						N
	English	Spanish	Portuguese	French	Other	Unknown	
USA	78.5%	6.2%	4.9%	0.6%	9.8%	0.1%	1,349
Brazil	68.3%	7.1%	13.9%	0.4%	10.4%	0.0%	482
UK	79.1%	6.1%	6.1%	1.8%	6.9%	0.0%	392
France	70.6%	7.8%	9.2%	3.9%	8.5%	0.0%	153
Other	73.6%	8.1%	8.6%	0.5%	9.2%	0.0%	568

We compare these differences using the models described above. The top panel in Table 3 displays the marginal probability of posting in English; the bottom panel displays the marginal probability of posting in Portuguese. Column headers indicate whether at least one of the authors is from the respective country. Within each author country, we show the relative probability of posting in English if the medium was Twitter (relative to Facebook). Standard errors are calculated using the delta method. Irrespective of author country, English is more likely to be used if the medium is Twitter relative to Facebook (Table 3, top panel; probabilities ranging between 7 to 21 percent and statistically significant at 5% significance).

**Table 3:** Probability to post in English or Portuguese on Twitter over Facebook, by Author’s Country.

*Panel A*: *Outcome in English*				
	**USA**	**UK**	**Brazil**	**France**
Probability of Posting in English on Twitter, Relative to Facebook	0.128 [0.105–0.150]	0.069 [0.028–0.110]	0.205 [0.163–0.247]	0.198 [0.125–0.271]
*Panel B*: *Outcome Portuguese*				
	**USA**	**UK**	**Brazil**	**France**
Probability of Posting in Portuguese on Twitter, Relative to Facebook	-0.045 [-0.056- -0.033]	-0.043 [-0.067- -0.019]	-0.125 [-0.156- -0.094]	-0.085 [-0.131- -0.039]
Num. observations	25,483	8,189	7,702	3,164

Note: Each column represents a separate model indicating author’s country of origin. 95% confidence intervals are shown in brackets and calculated using the delta method.

Table 4 displays results from Eq (2). Model [1] shows a reduced model where the variable β_1_ is dropped, showing the relative marginal probabilities of posting in English when some or all the authors are English irrespective of social media outlet. Model [2] shows results from Eq (2). Models [3] and [4] test for whether the estimated probabilities are sensitive to the number of authors on the paper. Model [3] includes number of author fixed effects, which is a vector of 31 indicator variables. (The number of authors on the papers included in our dataset ranges from 1 to 57, with 31 unique values.) Model [4] limits estimation to papers that have more than 2 authors.

**Table 4:** Probability posting in English, by author country English primacy.

	Model [1]		Model [2]		Model [3]		Model [4]	
Some authors English, relative to no authors English	0.020 [0.011–0.029]							
					
All authors English, relative to no authors English	0.016 [0.009–0.024]							
					
Some authors English, on Twitter, relative no authors English, on Twitter			-0.033 [-0.09–0.024]		-0.110 [-0.166- -0.054]		-0.032 [-0.09–0.026]	

All authors English, on Twitter, relative no authors English, on Twitter			-0.141 [-0.186- -0.096]		-0.159 [-0.209- -0.109]		-0.129 [-0.178- -0.08]	

Num. observations	44,980		44,980		42,851		32,206	

Note: Each column represents a separate model. 95% confidence intervals are shown in brackets and calculated using the delta method. Model [1] estimates probability of posting in English on Facebook or Twitter if some or all authors are from English-speaking countries, relative to having no authors from English speaking countries. Models [2], [3], and [4] estimate probabilities of posting in English on Twitter if some or all authors are from English-speaking countries, relative to posting in English on Twitter if no authors are from English speaking countries. Model [3] controls for the number of authors. Model [4] limits estimation to articles with two or more authors.

Results found in Table 4‘s Model [1] show that, on average, when some or all the authors are from primarily English-speaking countries, the probability of posting in English is greater but not by much (2 percent more likely and statistically significant). However, results from Model [2] show that the relative probability of posting in English when the medium is Twitter is less when some or all of the authors are from primarily English-speaking countries. These results are largely due to the fact that when some or all authors are from English speaking countries, both Twitter and Facebook posts are predominantly in English; conversely, when none of the authors are from English speaking countries, there is a much larger swing in the percentage posting in English on Twitter, relative to Facebook. Our preferred estimates are from Model [3], which control for correlations between the number of authors and the likelihood that more than one author will be from an English-speaking country, show that posting on Twitter is 11 percent less likely to be in English if some of the authors are from English speaking countries, compared to Twitter posts for papers in which no authors are from English speaking countries. When all of the authors are from English speaking countries, Twitter posts are 16 percent less likely to be in English compared to Twitter posts in which none of the authors are from English speaking countries.

**Fig 4:**
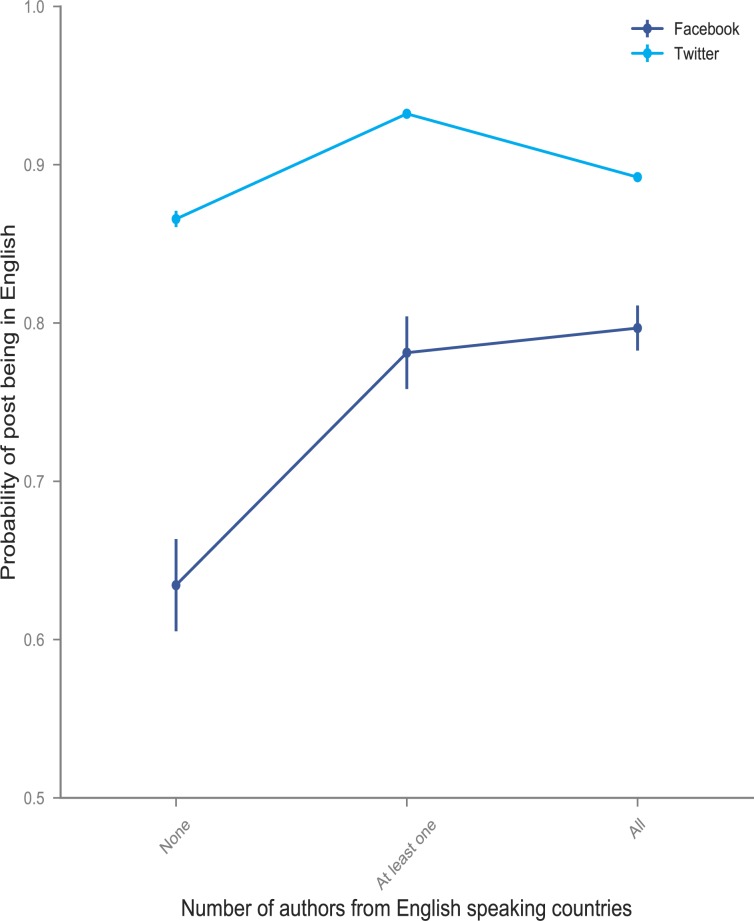
Probability of social media posts being in English when none, some or all authors from English-speaking countries.

We display the predicted probabilities from Eq (2), Model (3) in Fig 4. These predicted probabilities illustrate that posting in English on Facebook when no authors are from English-speaking countries is relatively rare (occurring about 65% of the time), but when some or all of the authors are from English-speaking countries, posting in English occurs much more frequently, slightly less than 80% of the time. When the medium switches to Twitter, posts in English occur more than 85% of the time, irrespective of author country of origin. Thus, the change in likelihood of posting in English from Facebook to Twitter is larger when none of the authors are from English-speaking countries.

## Discussion

Our analysis of the language of posts related to research articles on Twitter and Facebook indicate that both Twitter and Facebook are dominated by conversations in English (Fig 3, Table 1, Table 2). This corresponds to both the dominance of English in scientific publishing as well as the dominance of users from English speaking countries on both on Facebook and Twitter [32,33]. At the same time, the higher percentage of non-English posts on Facebook overall indicates that people, including non-English speakers, perceive the two platforms differently, with Twitter as a place for discussions with a global public and Facebook a place where more targeted (potentially locally relevant) discussions take place.

We examined local relevance by analyzing the country of the authors of each research paper. Our results show that researchers working outside of English-speaking contexts have their work taken up in non-English languages more than authors from English-speaking countries (Table 4). Even though nearly all of the papers were published in English, those papers authored with Brazilians, for example, were shared more often in Portuguese than papers from US and UK authors. We suggest that this local language effect may be an indication of researchers writing about national relevant aspects of Zika, or sharing it among their local colleagues and personal networks. However, when looking at the language of the conversations around research documents, the dominance of English is absolute, with both Spanish and Portuguese paling in comparison. Perhaps this is not surprising, given that the research has been mostly published in English, making the international scholarly community its main readers.

What is clear, given our analysis, is that, in spite of the dominance of English, there are significant differences in what language is used between the two platforms, and depending on who authored the papers being discussed. We find evidence of local interest in the research being conducted. The statistically significant differences indicate that there is something distinct about the kind of conversations that take place on the two platforms, something that is corroborated by the ‘local’ effect found on Facebook (where we found that the article author’s local language is the most likely to be used, after English). We hypothesize that two things are happening here: 1) that authors are researching topics that have national relevance, and 2) that Facebook, by its design, allows more local organization between users that gives them the liberty to communicate in their local language. However, without looking in more detail at the individuals who are behind the posts, it is impossible to determine if the differences between two platforms stem from different demographic of individuals, or from the same individuals choosing their local language when posting on Facebook and English on Twitter.

We try to gain a better understanding of the local and nationally relevant effect by paying special attention to Brazil, the country at the epicenter of the outbreak. Brazil, with a population of over 200 million Portuguese speakers, and a solid and historical know-how in tropical and neglected diseases, was the second most prolific country (after the US) in Zika research since the outbreak began [9]. Its research in tropical and neglected diseases is considered to be “outstanding” and growing at a rate twice the worldwide average [34]. Brazil’s strength and volume of output in Zika, along with the Brazilian governments pouring of resources into researching the virus [35], is indicative of a national interest in serving the local population.

For all these reasons, we might expect that if language is going to reflect the national relevant and local interest in research, it would be apparent in Brazil. We do, in fact, find that posts in English about research articles from Brazilian authors make up a smaller proportion posts, than on any of the other three most prolific countries, both Twitter and Facebook. On Facebook, where non-English is most likely, 14.2% of posts about papers with a Brazilian author are in Portuguese, 7.0% in Spanish and 10.3% in other languages Table 2). The platform effect is most pronounced for posts about papers with Brazilian authors, with posts on Facebook being 20.5% less likely to be in English than on Twitter, and 12.6% more likely to be in Portuguese (Table 3).

The combination of both these findings (the greater proportion of non-English and the increased likelihood of non-English on Facebook, relative to Twitter) in the case of Brazil, a country that was so obviously affected by the outbreak and that leads in research on tropical diseases, are a reflection and affirmation of the main findings of this study. Namely, that Twitter is a social media platform most often used in English by users who share research articles in English, suggesting that it is a more international arena to debate science and used with more academic/professional purposes while Facebook has a stronger presence of non-English languages, which might indicate its relevance as a national debate arena.

### Study limitations

Data referring to results on Facebook posts are known to be undercounted due to the way in which Altmetric LLC collects activity from that platform. Altmetric does not collect posts that take place on user’s pages (even when they are shared with the public), but rather only counts public posts that are made in pages (i.e., either groups or institutional pages). Considering the local role of Facebook, and the fact that it is user-to-user interactions that are undercounted, this limitation and its effect on understanding science communication deserves to be further investigated.

## Conclusion

The dominance of English as the language for research communication, both for research papers themselves, by authors from all countries, and on Twitter and Facebook is evident. This dominance should not be underestimated when seeking to communicate research to affected populations, or when seeking to do health surveillance on social media, especially when those affected populations are not in English-speaking countries. However, despite the dominance of English in the observed research communication, there are clear indications that there is interest in research about emerging outbreaks, like Zika, from non-English speakers.

The local effect we found (e.g., that work from Brazilian authors is most likely to be discussed in Portuguese and less likely to be discussed in English) suggests that researchers are pursuing nationally relevant research topics (perhaps guided by national funding mandates, or simply from personal interest). Whatever the reason, if the research from such authors is resonating with local populations, the Zika outbreak example shows us that it may be desirable to open up formal channels of research communication in languages other than English.

Similarly, the significant differences between Twitter and Facebook, present across all author countries, shows us that not all social media outreach should be treated equally. The language differences found point to either distinct populations or different cultural practices on the two platforms, both of which warrant consideration for science communicators or those seeking to do health surveillance. In particular, our findings regarding the Zika outbreak point to the need to give serious consideration to the role of Facebook if the intent is to help information reach affected populations in countries like Brazil.

Finally, we believe our work also highlights that academic journals are an important source of primary information, not only for scholars, but also for the general public that might come across research while searching for information in the internet, especially when those journals make their content freely available through open access. While our analysis did not study if those sharing Zika-related research were academics or not, the use of Facebook as a platform for sharing in languages other than English shows us that journals would be able to contribute further for the social impact of research if they considered outreach activities aimed at the international community in their native languages.

## Supporting information

S1 Appendix Additional analysis by journal country of publication. (DOCX)
