# Supplementary material for: Local chatter or international buzz? Language differences on posts about Zika research on Twitter and Facebook
Source: PLoS One. 2018 Jan 5;13(1):e0190482. doi: 10.1371/journal.pone.0190482 (PMC5755770; doi:10.1371/journal.pone.0190482)
Supplement: S1 Appendix — (DOCX) [file pone.0190482.s001.docx]

Supporting Information

**S1 Appendix. Additional analysis by journal country of publication.**

We also estimate a comparable model but subset the data by the journal’s origin country (as opposed to the author’s origin country). We exclude France from this list as only 16 posts are available when the journal’s country of origin is France. Regardless of the journal’s origin country, the marginal probability of posting in English is greater if the medium was Twitter (probabilities between 15 and 54 percent and statistically significant) (S1 Table).

S1 Table. Probability post in English or Portuguese, by Journal Country

|  | **USA** | **UK** | **Brazil** |
| --- | --- | --- | --- |
| Probability of Posting in English on Twitter, Relative to Facebook | 0.147  [0.125-0.168] | 0.123  [0.09-0.156] | 0.536  [0.278-0.794] |
|  | **USA** | **UK** | **Brazil** |
| Probability of Posting in Portuguese on Twitter, Relative to Facebook | -0.056  [-0.068- -0.044] | -0.072  [-0.093- -0.05] | -0.537  [-0.806- -0.269] |
| Number of observations | 28,868 | 14,631 | 154 |

Note: Each column represents a separate model indicating author’s country of origin. 95% confidence intervals are shown in brackets and calculated using the delta method.
